# *Aspergillus nidulans* Cell Wall Composition and Function Change in Response to Hosting Several *Aspergillus fumigatus* UDP-Galactopyranose Mutase Activity Mutants

**DOI:** 10.1371/journal.pone.0085735

**Published:** 2014-01-15

**Authors:** Md Kausar Alam, Karin E. van Straaten, David A. R. Sanders, Susan G. W. Kaminskyj

**Affiliations:** 1 Department of Biology, University of Saskatchewan, Saskatoon, Saskatchewan, Canada; 2 Department of Chemistry, University of Saskatchewan, Saskatoon, Saskatchewan, Canada; Universidade de Sao Paulo, Brazil

## Abstract

Deletion or repression of Aspergillus nidulans ugmA (AnugmA), involved in galactofuranose biosynthesis, impairs growth and increases sensitivity to Caspofungin, a β-1,3-glucan synthesis antagonist. The A. fumigatus UgmA (AfUgmA) crystal structure has been determined. From that study, AfUgmA mutants with altered enzyme activity were transformed into AnugmA▵ to assess their effect on growth and wall composition in A. nidulans. The complemented (AnugmA::wild type AfugmA) strain had wild type phenotype, indicating these genes had functional homology. Consistent with in vitro studies, AfUgmA residues R182 and R327 were important for its function in vivo, with even conservative amino (RK) substitutions producing AnugmA? phenotype strains. Similarly, the conserved AfUgmA loop III histidine (H63) was important for Galf generation: the H63N strain had a partially rescued phenotype compared to AnugmA▵. Collectively, A. nidulans strains that hosted mutated AfUgmA constructs with low enzyme activity showed increased hyphal surface adhesion as assessed by binding fluorescent latex beads. Consistent with previous qPCR results, immunofluorescence and ELISA indicated that AnugmA▵ and AfugmA-mutated A. nidulans strains had increased α-glucan and decreased β-glucan in their cell walls compared to wild type and AfugmA-complemented strains. Like the AnugmA▵ strain, A. nidulans strains containing mutated AfugmA showed increased sensitivity to antifungal drugs, particularly Caspofungin. Reduced β-glucan content was correlated with increased Caspofungin sensitivity. Aspergillus nidulans wall Galf, α-glucan, and β-glucan content was correlated in A. nidulans hyphal walls, suggesting dynamic coordination between cell wall synthesis and cell wall integrity.

## Introduction

The cell wall is essential for the survival of fungi in natural environments. In fungi, the cell wall is about one quarter of the average fungal biomass [1], and about a third of the fungal genome (∼4000 genes) is involved in cell wall biosynthesis and maintenance [2]. Polysaccharides and glycoconjugates contribute to proper functioning of the cell wall and cell membranes that collectively form the interface between the fungal cell and its environment [3]. Due to the importance of cell walls for fungal growth and host invasion, and the relative lack of conservation with animal cell components, the cell wall is expected to be a good source of potential antifungal targets [4], [5].

*Aspergillus fumigatus* is considered to be the most important airborne, human pathogenic fungus [6]. *Aspergillus nidulans* is a closely related model species with easy molecular genetic manipulation [7]. Carbohydrate analysis of *A. fumigatus* and *A. nidulans* walls shows that both species have similar composition [8], ∼40% each of α-glucan and β-glucan [1], [9]. Despite decades of research characterizing fungal cell wall composition and structure [2], [5], many details remain poorly understood. Notably, recent evidence shows that fungal walls can be rapidly remodeled in response to environmental change [10], adds another layer of complexity.

Galactofuranose (Gal*f*) has an important role in fungal wall maturation [11]–[14]. Gal*f* is found in glycoconjugates that are important for survival or virulence of microorganisms including some bacteria, protozoa and fungi [15]–[19], but it is lacking in vertebrates. Since Gal*f* is absent from higher eukaryotes and is involved in growth or virulence of some bacteria and fungi, the enzymes involved in its biosynthesis are potential drug targets [19], [20]. Notably, loss of or reduction in wall Gal*f* following gene deletion or repression in *A. nidulans* correlated with increased caspofungin sensitivity *in vitro* [12], [13]. *In vivo* pathogenicity studies related to wall Gal*f* are few, and to date are equivocal [15], [18]. We have characterized the Gal*f* biosynthesis pathway in *A. nidulans* as a model system [11]–[14], [21] and are extending this to *A. fumigatus* in preparation for future testing of antifungals.

UDP-galactopyranose mutase is the first committed enzyme in the Gal*f* biosynthesis pathway in both *A. fumigatus* (*Af*GlfA [18] also called *Af*UgmA [15]) and *A. nidulans* (*An*UgmA [11]). *Aspergillus nidulans* strains deleted for An*ugmA* have 500-fold reduced hyphal growth and spore production [11]. UgmA is unique in *A. nidulans* and *A. fumigatus*, but is not essential in either species.

The crystal structure of *Af*UgmA has been determined [22]. *Af*UgmA has conserved arginine residues, R182 and R327 that are important for function *in vitro*. In addition, H63 and F66 are expected to contribute to *Af*UgmA activity because the loop containing them changes position depending on the redox state of the FAD cofactor [22]. Assessing the roles of these amino acids for the *in vivo Aspergillus* phenotype is expected to refine the *in vitro* characterization of UGM [22], since *in vitro* enzymatic studies might not be precisely comparable to their *in vivo* function [23], and because H63N mutant did not express sufficiently for *in vitro* characterization. Here we examined the *in vivo* effects of structurally and functionally important amino acid residues of *Af*UgmA using *A. nidulans* as a host system, comparing cell wall composition, hyphal surface adhesion and response to antifungal drugs.

## Results

We used *A. nidulans* as a host for wild type (WT), wild type complemented (WC), and single amino acid mutants in *Af*UgmA to assess their effect on colony growth, wall composition, wall surface adhesiveness, and drug sensitivity. We hypothesized there would be concordance between *Af*UgmA function *in vitro* and the *in vivo* phenotype; *in vivo* characterization provided additional information.

### Fungal phenotype for mutations affecting *Af*UgmA activity

**Figure 1:**
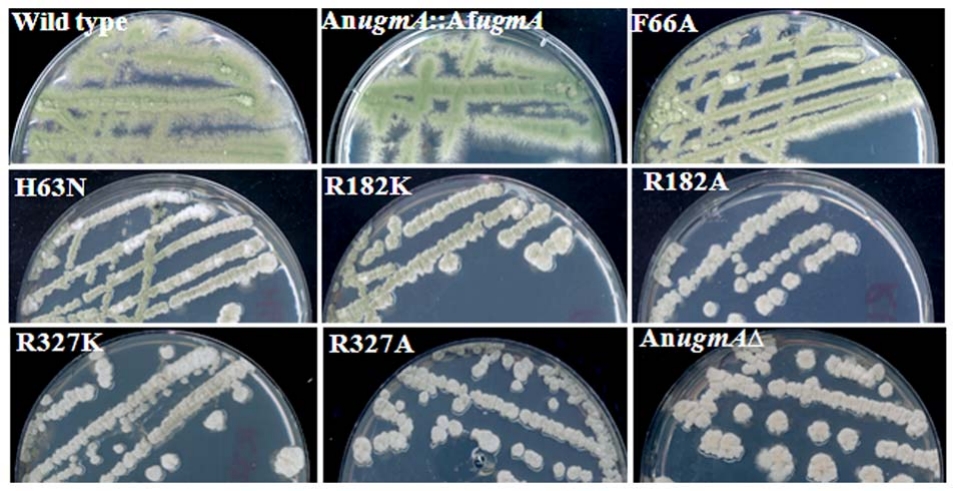
Colony morphology of *Aspergillus nidulans* wild type (WT) strain complemented with wild type *Af*UgmA (WC), single residue *Af*UgmA mutants (F66A, H63N, R182K, R182A, R327K, R327A) and An*ugmA*Δ strains, grown on complete medium at 30°C for 3 d. The colour difference between WT and WC strains was due to slightly different ages of culture. See Figure S1 in File S1 for a direct comparison of the spore colours of these two strains.

We generated an [An*ugmA*:: wild type Af*ugmA*] complemented (WC) strain in *A. nidulans* to confirm functional homology, and for quantitative characterization that had not been done previously [11]. The WC strain phenocopied wild type *A. nidulans* hyphal morphometry and colony development (Figure 1, Table 1, and Figure S1 in File S1). Table 2 presents the relative enzyme activity of wild type and mutated UgmA strains, derived from data in Table 3 of [22]. As expected, the R327K and R327A strains phenocopied the An*ugmA*Δ strain for spore formation, colony growth, and hyphal morphometry (Figure 1, Table 1). Reduced sporulation was due to fewer conidiophores and aberrant conidiophore formation (Figure S2 in File S1), comparable to the An*ugmA*Δ strain. The R182K *in vivo* phenotype had improved sporulation but not colony growth compared to An*ugmA*Δ. There were no significant differences between the R182A and An*ugmA*Δ phenotypes (Figure 1, Table 1). Sporulation in *A. nidulans* appears to have a less stringent requirement for Gal*f* content than does hyphal growth (Table 2).

**Table 1:** Colony and hyphal characteristics^a^ of wild type, and An*ugmA*▵ strains complemented with wild type AfUgmA, single residue AfUgmA mutants and deletion strains grown on CM.

Strains	Spores/colony (×10^6^)^b^	Colony diameter (mm)^c^	Hyphal width (µm)^d^	Basal cell length (µm)^d^
Wild type (A1149)	109±3.0^e^	18±0.5^e^	2.5±0.1^e^	29±1.7^e^
An*ugmA*▵:Af*ugmA*	99±2.3^f^	17±0.6^e^	2.5±0.1^e^	27±1.7^e^
F66A	90±2.8^g^	13±0.3^f^	2.6±0.1^e^	24±0.9^e^
H63N	6.4±0.4^h^	7.3±0.4^g^	3.3±0.1^f^	18±0.8^f^
R182K	3.2±0.2^i^	6.2±0.3^h^	3.4±0.1^fg^	17±1.8^f^
R182A	1.3±0.1^j^	5.0±0.3^i^	3.5±0.1^gh^	16±0.7^fg^
R327K	1.2±0.1^j^	5.1±0.3^i^	3.5±0.1^ghi^	15±0.8^g^
R327A	0.6±0.1^k^	4.6±0.4^i^	3.6±0.2^hi^	14±0.8^g^
An*ugmA*▵	0.5±0.4^k^	5.2±0.4^i^	3.7±0.1^hi^	14±0.8^g^

a Values are presented as mean ± standard error. Statistical significance of the mean values was compared using a Kruskal–Wallis test, and shown for each characteristic to be significantly different at P<0.05. For each column, values followed by different letters (e–k) are significantly different at P<0.05.

b At least 4 colonies per strain were counted. Spores of wild type and mutated strains were streaked on CM and incubated for 3 d at 28°C to give isolated colonies.

c The diameter of ten colonies/strain was measured using a dissection microscope.

d Hyphal width was measured at septa. Basal cell length was between adjacent septa for n = 25 measurements per strain.

**Table 2:** *Aspergillus nidulans* wild type and chimaera cell wall carbohydrates^a^, antifungal drug sensitivity^ac^, and relative UGM activity^b^.

Strains	Relative UgmA	Gal*f* a	α-glucan a	β-glucan a	Antifungal drug sensitivity^a^		
	activity^b^				Casp^c^	Itra^c^	CFW^c^
Wild type	1	1	1	1	1	1	1
An*ugmA* :: Af*ugmA*	nd	0.96	1.05	0.96	1	1	1
F66A	0.16	0.78	1.18	0.87	1.2	1	1.1
H63N	na	0.52	1.46	0.74	1.5	1.1	1.1
R182K	0.10	0.43	1.58	0.69	1.7	1.2	1.2
R182A	6.0×10^−6^	0.13	1.68	0.60	1.5	1.2	1.1
R327K	0.015	0.09	1.70	0.60	1.9	1.2	0.9
R327A	bd	0.04	1.79	0.58	1.7	1.2	1.1
An*ugmA*▵	nd	0.00	1.84	0.57	1.9	1.2	1

^a^ Absorbance at 405 nm was recorded using ELISA reader, Results are presented as an index of OD values with respect to wild type. Cell walls were isolated from *A. nidulans* strains after 48 h growth. Index sensitivity values are for each drug/strain combination compared to wild type. Index values > 1.0 were more sensitive than wild type. Index values that differed by > 0.2 were based on data that were significantly different.

^b^ Relative UGM activity *in vitro* with respect to wild type, derived from data in (33) and this study for F66A. nd, not determined; na, not available (low protein expression); bd, below detection.

^c^ Caspofungin, Casp; Itraconazole, Itra; Calcofluor White, CFW. See Materials and Methods for drug dosage and medium formulation. Drug sensitivity was measured using a disc diffusion assay as described in methods (Figure S3 in File S1). For Caspofungin, sensitivity was the radius (mm) of the clear zone with no visible growth. Mean ± SE of two measurements for each of four biological replicates were used for statistical analysis (not shown).

H63 is expected to contribute to *Af*UgmA activity because it is part of the flexible loop (loop III) above the *si*-face of the isoalloxazine ring that changes position due to the redox state of the FAD cofactor [22]. Structural analysis suggests H63N might keep UgmA loop III in the conformation similar to that seen in prokaryotic UGMs [24]–[26] and the reduced *Af*UgmA structure [22] by forming an H-bond with Q458. The *in vitro* H63N enzyme activity was not tested because of low protein expression for this construct. Notably, the *in vivo* H63N phenotype was comparable to that of R182K (Table 1, Figures 1 and Figure S2 in File S1), implying that the H63N strain likely would show a similar enzyme activity to R182K. This suggests that the flexibility of loop III plays a role in the catalytic activity of *An*UGM. As with R182K, the major improvement compared to An*ugmA*Δ was in sporulation. This is consistent with Gal*f* having multiple roles in the *Aspergillus* phenotype, with sporulation being more responsive to low levels of UgmA activity than hyphal morphology.

F66 is at the end of *Af*UgmA loop III and may control loop III flipping and consequently opening of the mobile loops depending on the redox state of the cofactor [22]. The *Af*UgmA-F66A *in vitro* enzyme activity was almost double that of R182K (Table 2). *In vivo*, the F66A phenotype was relatively comparable to wild type (Table 1, Figure 1) regarding sporulation (Figure S2 in File S1) and hyphal morphometry, but restricted for colony diameter.

Together, these data show that *Af*UgmA R327 is critical for function *in vitro* and *in vivo*. H63, F66, and R182 each contribute to catalytic efficiency but to a much lesser extent. For all mutants, sporulation was less dependent on UgmA activity than hyphal or colony morphometry, consistent with the fact that *ugmA▵* and R327A strains had limited sporulation. In sum, the *in vivo Af*UgmA mutant phenotypes correlated well with *in vitro* UgmA activity, and for H63N the *in vivo* data extended otherwise unobtainable characterization.

### Loss of wall Gal*f* is associated with increased hyphal surface adhesion

**Figure 2:**
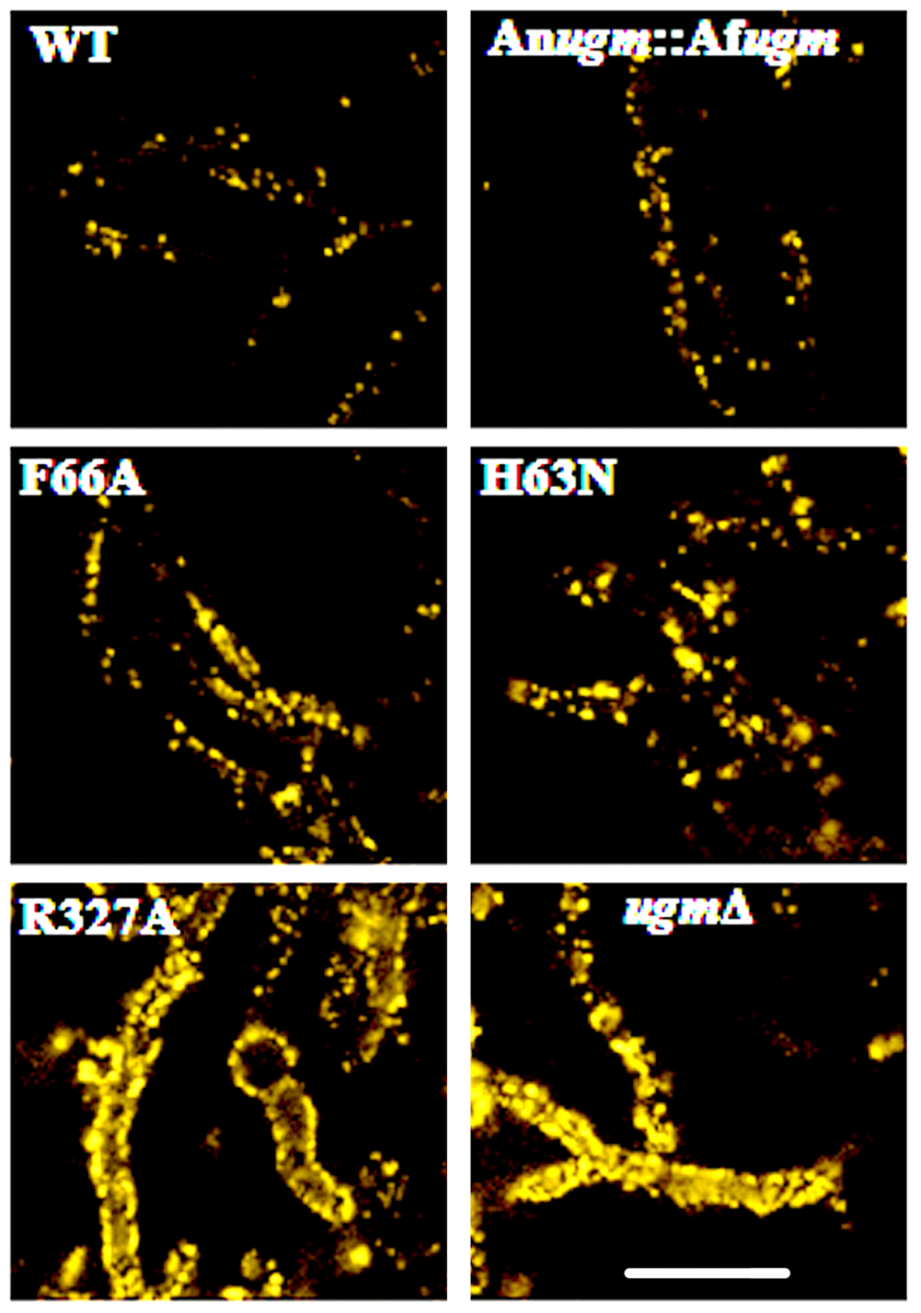
Surface adhesion of *Aspergillus nidulans* strains to fluorescent latex beads. Wild type (WT), strain complemented with wild type *Af*UgmA (WC), single residue *Af*UgmA mutants (F66A, H63N and R327A) and An*ugmA*Δ strains. Bar  =  20 µm is for all images.

We used fluorescent latex beads visualized with confocal microscopy to assess hyphal surface adhesiveness, as adapted from [13]. The WT and WC hyphae had relatively fewer fluorescent beads attached compared to An*ugmA▵.* Bead attachment for the strains with R182K, R182A, R327K, or R327A was similar to An*ugmA▵* (Figure 2). Figure 2 used bead adhesion by the R327A strain to represent all of these latter mutant strains. For the F66A and H63N strains, bead attachment was slightly higher than wild type or WC strains but much lower than An*ugmA▵* or the other mutant strains (Figure 2). The bead adhesion assay showed major changes in adhesion, but did not discriminate between subtle differences. Atomic force microscope (AFM) force mapping might reveal these changes, but this is currently beyond our scope. However, our previous published paper showed using AFM force mapping that *ugmA*Δ strain hyphal wall surfaces had higher adhesion and reduced resilience compared to wild type *A. nidulans* walls [21].

### *Af*UgmA active site mutations do not affect UgmA-GFP cytoplasmic distribution

**Figure 3:**
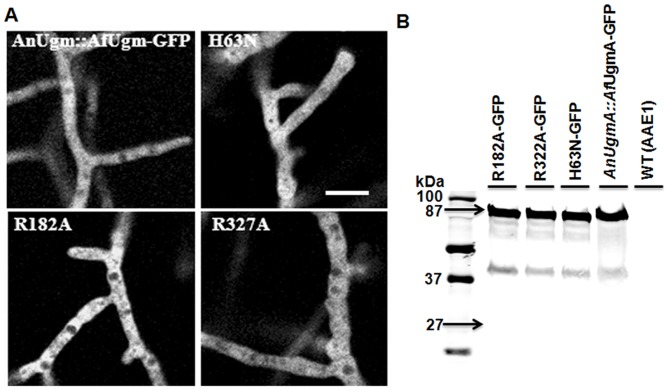
*In vivo* distribution of GFP-tagged *Af*UgmA in *Aspergillus nidulans*. A. Wild type complemented (WC) and single residue mutants (H63N, R182A and R327A) have comparable *Af*UgmA-GFP distribution. The single residue mutants have the *ugmA*Δ hyphal morphology. Bar  =  20 µm for all images. B. Confirmation of *Af*UgmA-GFP fusion protein distribution by Western blot. Total protein was extracted from *A. nidulans* wild type (WT; AAE1) and GFP-tagged *Af*UgmA strains (R182A-GFP, R327A-GFP, H63N-GFP, *An*UgmA::*Af*Ugm-GFP). Total Proteins (15 µg/lane) were separated on 10% SDS-PAGE and immunoblotted with an anti-GFP antibody.

El-Ganiny *et al.* [14] showed that *An*UgmA-GFP was cytoplasmic and evenly distributed along hyphae, consistent with UgmA lacking a signal peptide (also suggested in [15]). To confirm that expression of wild type and mutated *Af*UgmA did not affect cytoplasmic distribution in *A. nidulans*, strains were C-terminal GFP-tagged under the control of the *ugmA* endogenous promoter (Figure S3 in File S1). Previously, we had found that N-terminal *An*UgmA-GFP abolished wild type UgmA function, since an RFP-UgmA strain had an An*ugmA▵* phenotype (El-Ganiny and Kaminskyj, *unpublished*). The WC-GFP strain had WT hyphal and colony morphology, and abundant conidiation (*data not shown*), indicating that *Af*UgmA-GFP did not interfere with *A. nidulans* growth. The *A. nidulans* strains containing GFP-tagged R182A, R327A or H63N had compact colony growth, phenotypically similar to the respective untagged strains. GFP distribution in each strain was cytosolic, excluded from membrane-bounded organelles, and lacked a pronounced longitudinal gradient (Figure 3A), consistent with wild type distribution of *An*UgmA in *A. nidulans* [14].

To confirm that the even GFP distribution was not due to cleavage of the GFP tag, we extracted total proteins from the wild-type and mutated GFP-tagged strains (WT, R182A-GFP, R327A-GFP, H63N-GFP and *Af*UgmA-GFP) strains, followed by separation of proteins using 10% SDS-PAGE and western blotting with anti-GFP (Figure 3B). Notably, the great preponderance of signal was at 87 kDa, consistent with GFP-tagged UgmA, and essentially none was at 27 kDa, which would be consistent with cleaved GFP. The anti-GFP western blotted protein preparations from GFP-tagged mutant strains (Figure 3B) were consistent with wild type AnUgmA-GFP distribution [14] and with *Af*UgmA-GFP distribution (Figure S4 in File S1). This suggested that the phenotype effects of mutated *Af*UgmA constructs did not relate to alterations in sub-cellular distribution. We do not have an anti-UgmA, so we were unable to perform the complementary experiments to directly measure the intracellular UgmA levels *in vivo* in order to confirm the stability of *Af*UgmA.

### Alteration in Gal*f* affects wall glucan composition in *A. nidulans*

**Figure 4:**
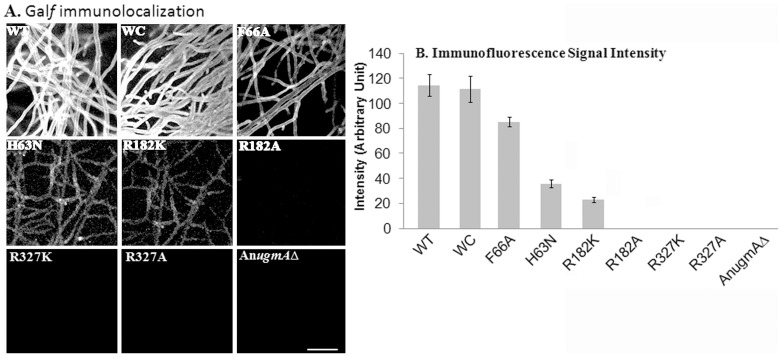
Localization of Galactofuranose (Gal*f*) in *Aspergillus nidulans* Cell wall. A. Gal*f* immunolocalization. B. Immunofluorescence quantification of Gal*f* using confocal system software (see Methods). Error bar shows standard error. *Aspergillus nidulans* wild type (WT), wild type *Af*UgmA-complemented (WC), mutated *Af*UgmA (as listed), and An*ugmAΔ*. Bar  =  10 μm for all images.

**Figure 5:**
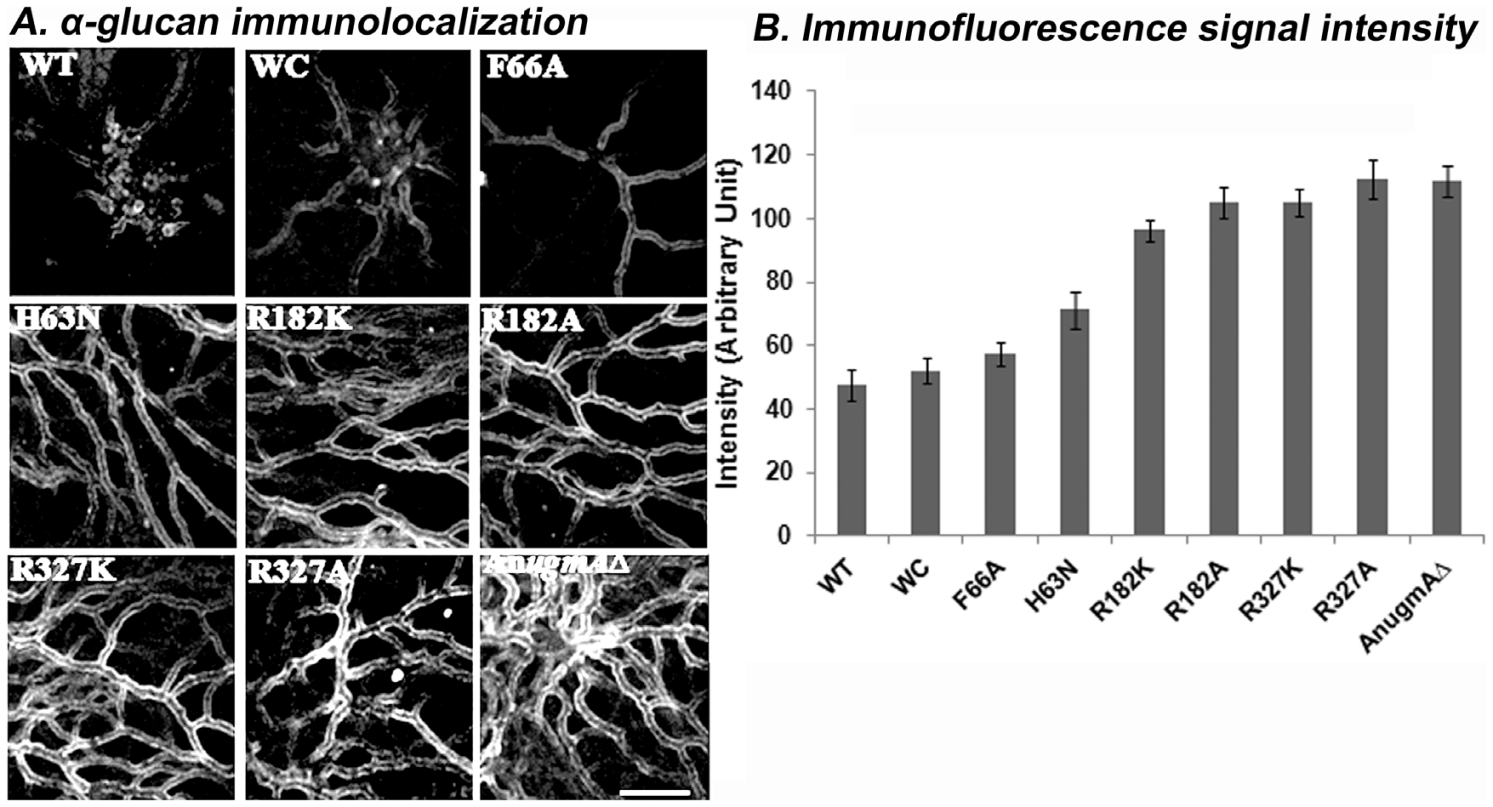
Localization of Alpha-glucan in *Aspergillus nidulans* Cell wall. A. Alpha-glucan immunolocalization. B. Immunofluorescence quantification of Alpha-glucan using confocal system software (see Methods). Error bar shows standard error. *Aspergillus nidulans* wild type (WT), wild type *Af*UgmA-complemented (WC), mutated *Af*UgmA (as listed), and An*ugmAΔ*. Bar  =  10 μm for all images.

**Figure 6:**
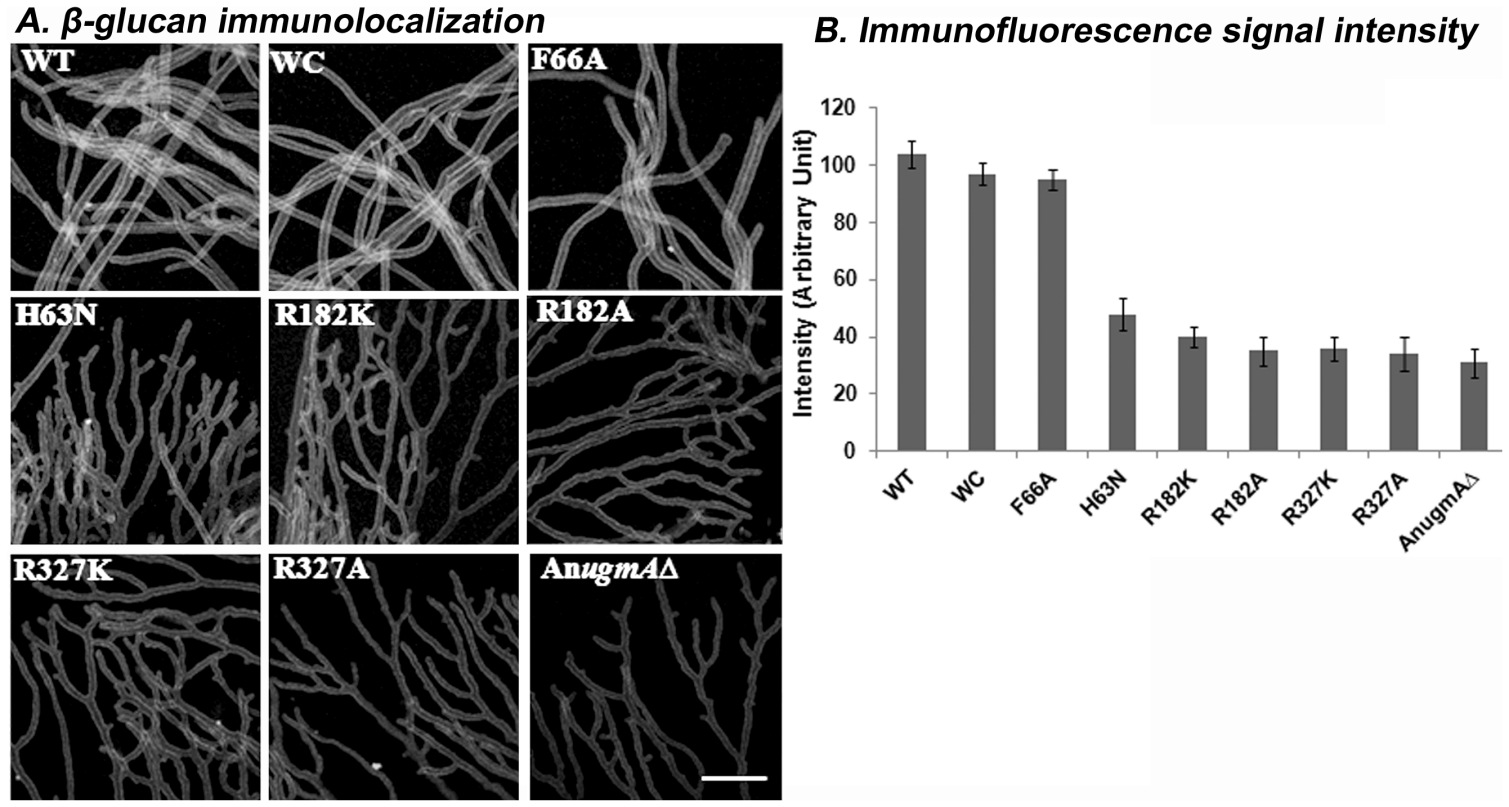
Localization of Beta-glucan in *Aspergillus nidulans* Cell wall. A. Beta-glucan immunolocalization. B. Immunofluorescence quantification of Beta-glucan using confocal system software (see Methods). Error bar shows standard error. *Aspergillus nidulans* wild type (WT), wild type *Af*UgmA-complemented (WC), mutated *Af*UgmA (as listed), and An*ugmAΔ*. Bar  =  10 μm for all images.

Alam *et al.* 2012 [13] showed using qPCR that gene expression of α-glucan synthase, *agsB* and β-glucan synthase, *fksA* were influenced by the level of An*ugmA* activity. However, synthase gene expression is not necessarily directly related to wall glucan content. We quantified cell wall α-glucan, β-glucan, and Gal*f* using ELISA (Table 2) and immunofluorescence (Figures 4–6) in WT, WC, mutated and An*ugmA▵* strains.

*Aspergillus nidulans* WT and WC strains had strong Gal*f* immunolocalization in hyphal walls (Figure 4). The F66A strain had the highest level of hyphal wall Gal*f* immunofluorescence of any of the mutants, consistent with ELISA quantification (Table 2) and with its relatively high *in vitro* UgmA activity (Table 2). The R182K and H63N strain hyphal wall Gal*f* immunofluorescence quantitation was half that of F66A (Figure 4), comparable to ELISA results (Table 2) showing Gal*f* content in these strains is 35–45% of F66A. Low hyphal wall Gal*f* was consistent with phenotypic differences, showing concordance between wall composition and hyphal morphology (Figure 1, Tables 1, 2). WT, WC and F66A cell wall Gal*f* content in (Table 2) was correlated with sporulation (Table 1, Figs. 1, S2 in File S1). The R327K, R327A, R182A strains lacked immunolocalizable Gal*f* and all aspects of their phenotype resembled the An*ugmA*Δ strain. Taken together, wall Gal*f* content was consistent with *in vitro Af*UgmA activity and with other aspects of hyphal and colony development phenotype.

*Aspergillus nidulans* wild type and WC hyphal walls had relatively low α-glucan content (Table 2, Figure 5) compared to mutant and An*ugmA*Δ strains. This is consistent with our previous qPCR studies on gene expression [13], but was more nuanced regarding cellular distribution. Alpha-glucan staining was most pronounced in the older hyphal regions, and was undetectable at wild type and WC hyphal tips. The α-glucan immunofluorescence intensities were stronger in the R327K, R327A, R182K, R182A and An*ugmA▵* strains (Figure 5), but again were substantially lower at hyphal tips (*not shown*). ELISA results for α-glucan (Table 2) were comparable to immunofluorescence quantification in older regions of the hyphae. In sum, wall α-glucan content of all the mutants and An*ugmA▵* strains was influenced by alteration in Gal*f* and it was inversely correlated with UgmA enzyme activity levels (Table 2).

*Aspergillus nidulans* wild type and WC hyphal walls had relatively higher β-glucan levels than the mutants and An*ugmA*Δ strains (Figure 6, Table 2). The quantitative difference in β-glucan level between WT, WC, F66A and all other strains was more pronounced than for α-glucan or Gal*f*. ELISA results for β-glucan were consistent with immunofluorescence quantification, showing a decline in wall β-glucan content that correlated with decrease in UgmA enzyme activity levels (Table 2).

These data show the relative importance of *Af*Ugm amino acid residues in and near the active site is R327 > R182 ∼ H63 >> F66. Clearly, *in vitro* enzyme and *in vivo* phenotype analysis have strong complementarity. We plan to use phenotype analysis as part of our strategy to assess the efficacy of potential UgmA-inhibitor compounds.

### Loss of *Af*UgmA activity leads to increased sensitivity to some antifungal compounds

If Gal*f* biosynthesis is to achieve its promise as an antifungal drug development target, most likely as a part of a combination therapy, strains with reduced *Af*UgmA should be expected to be more sensitive to antifungal compounds. We compared Caspofungin (β-glucan synthesis), Itraconazole (ergosterol synthesis), and Calcofluor White (chitin crystallization) sensitivity on all the strains (Table 2 and S7 in in File S1). Analysis of the raw drug sensitivity data showed that index values that differed by 0.2 were significantly different, as it had been seen in our previous studies [13]. None of the mutations notably affected sensitivity to Calcofluor White. Strains with substantially reduced Gal*f* (R182 and R327 mutants) were significantly more sensitive to Itraconazole. Furthermore, Caspofungin sensitivity was significantly increased for all of the mutant strains compared to wild type, almost 2-fold for R327K and *AnugmA*Δ. Even F66A, which still had a substantially wild type hyphal and colony phenotype, and wall composition was significantly more sensitive to Caspofungin (Table 2).

## Discussion

We used quantitative methods to correlate *in vitro* UgmA enzymatic function and *in vivo* fungal cell and developmental phenotype related to site-directed changes in conserved amino acid residues in the *Af*UgmA catalytic site. We were able to assess residues for which there was structural and *in vitro* enzymatic function information [22], as well as residues where enzymatic activity had not yet been assessed. These analyses provide a coherent picture of changes in *A. nidulans* related to the efficacy of an enzyme involved in an early step in Gal*f* biosynthesis in *Aspergillus* that affects wall composition-structure-function.

Previously we showed qualitatively that *A. fumigatus* Af*ugmA* restored wild type hyphal morphology in the *A. nidulans ugmA*Δ strain [11]. We have extended that preliminary observation with quantitative assessment of colony phenotype and wall composition for *A. nidulans* whose native *An*UgmA had been replaced with wild type *Af*UgmA or with *Af*UgmA constructs mutated in or near the enzyme active site. This approach provided a way to correlate structure and function of *Af*UgmA, regardless of whether gene products could be analyzed *in vitro*. According to van Straaten *et al.* [22], for *in vitro* enzymatic studies where all the mutants (except H63N) were over-expressed, purified and studied, there were no apparent changes in protein stability. There was no evidence of changes to the overall protein structure since the mutants crystallized under similar conditions and resulted in similar crystallographic structures [22]. In addition, none of the mutations affected the cellular *Af*UgmA distribution assessed with GFP-tagging and anti-GFP western blotting. These results are consistent with the effects we observed being due to changes in *Af*UgmA activity or other aspects of cellular interaction. Since all the mutants were expressed under endogenous An*ugmA* promoter to assess phenotype *in vivo*, it is also possible that these effects might be due to the instability of mutant protein. Although western blot showed the same level of fluorescence intensity for GFP tagged mutants and wild type *Af*UgmA against anti-GFP antibody, direct intracellular evaluation of these mutants and wild type *Af*UgmA stability *in vivo* is beyond our scope at this moment because of lacking an antibody directed against *Af*UgmA.

Comparing protein structure analysis and *in vitro* enzymatic activity of wild type *Af*UgmA with conservative (RK) and non-conservative (RA) mutants showed that R327 was essential to UgmA function [22] *in vivo*. Structural data in [22] shows that R327 stabilizes the position of the diphosphates of the nucleotide sugar and facilitates positioning of the galactose for catalysis. The R327K mutation impaired enzyme function whereas the R327A mutation produced a strain with no UgmA activity detectable *in vitro* (Table 2, and [22]). Consistent with this, *A. nidulans* strains whose wild type *An*UgmA had been replaced with AfUgmA-R327A qualitatively and quantitatively resembled the An*ugmA*Δ strain. The role of R182 was slightly less critical than that of R327 (Table 2). R182 is important for sugar orientation related to catalysis, as well as for catalytic efficiency [22]. When *Af*UgmA-R182K replaced the native *An*UgmA, the R182K strain showed significantly better growth and sporulation than An*ugmA*Δ, whereas the R182A strain resembled the An*ugmA*Δ deletion strain.

This is the first time that we have been able to partially rescue the An*ugmA*Δ phenotype by genetic means. Previously we had shown that low levels of Calcofluor White, or 1 M sucrose, partially remediated the An*ugmA*Δ defects, however this was only for morphogenesis of submerged hyphae, and not for sporulation [11]. Similarly, the R182K strain showed that sporulation was more effectively remediated by low levels of Gal*f* than was hyphal morphogenesis. Gal*f* immunolocalization using EBA2 [11] vs L10 [13] showed substantially different localization patterns. EBA2 preferentially stained conidia, metulae and phialides, whereas L10 stained hyphae. Together these suggest that there are at least two different types of Gal*f*-containing compound in *Aspergillus* walls.

We also examined the roles of two amino acids that could be functionally important because they are part of loop III, which moves upon redox state of the cofactor [22]. H63 is a highly conserved residue for prokaryotic and eukaryotic UGMs. The H63N construct expressed poorly *in vitro* so it had not been studied for *in vitro* enzyme activity. Histidine is positively charged at physiological pH whereas asparagine is polar but not charged. Correlating strain phenotype characteristics with estimates of UgmA activity suggested that the H63N strain UgmA activity would be comparable with the R182K strain. F66 is a highly conserved residue amongst eukaryotic UGMs. The *Af*UgmA F66A strain showed a 6.3-fold decrease in UgmA activity compared to wild type *Af*UgmA *in vitro* (Table 2). The *in vivo* F66A phenotype was similar to wild type, indicating that *in vivo* function of UgmA does not impair colony morphogenesis at this level of decrease *in vitro* activity. Our results further indicate that changes in *Af*UgmA loop III affect catalytic activity and that H63 is important but not critical for UGM activity.

Our previous work showed that deletion or down-regulation of any of three sequential genes in *A. nidulans* Gal*f* biosynthesis was associated with comparable reductions in hyphal growth rate and sporulation [11]–[14], with increased sensitivity to fungal wall-targeting compounds [12], [13], with changes in α-glucan and β-glucan synthase gene expression [13], and with changes in wall surface adhesion [21]. Here we have correlated the effect of mutations in *Af*UgmA (transformed into *A. nidulans*) with morphometry, with quantitative immunofluorescence, with ELISA quantification of wall carbohydrates, with changes in wall adhesiveness, and with sensitivity to anti-fungal compounds.

Notably, reductions in wall Gal*f* content correlated with increased wall α-glucan content (r^2^ = 0.972, P = 0.0001) and in decreased wall β-glucan content (r^2^ =  0.980, P = 0.0001) shown in this study were consistent with increased *agsB* and decreased *fksA* expression [13]. Not surprisingly, *A. nidulans* cell wall α-glucan and β-glucan content were also strongly correlated (r^2^ = 0.993, P<0.0001). Given that in wild type cells α-glucan and β-glucan are each thought to comprise about 40% of the *A. nidulans* wall [1], [9], this strongly suggests that the cell is metabolically constrained as to where it apportions metabolites for wall synthesis, so that increases in abundance of one major polymer must limit the resources available for synthesis of the others. However, this does not directly explain why engineered changes in Gal*f* content (perhaps 5% of total [15]) are so strongly related to changes in α-glucan and β-glucan content. We suspect these will be coordinated through the cell wall integrity pathway, which is a focus of current research.

Decreased *fksA* expression and β-glucan content correlated with increased sensitivity to Caspofungin (r^2^ = 0.855, P<0.0005), which is mechanistically satisfying. Notably, a *ugmA* overexpressed strain also showed Caspofungin hypersensitivity [13] suggesting that balanced expression of wall components is important for wild type phenotype and drug resistance.

The mutant (except F66A) and deletion strains also showed smaller but still significant increases in sensitivity to Itraconazole. Previously using AFM force mapping [21] we had found that *ugmA*Δ strain wall surfaces were more force-compliant than wild type walls, suggesting that they had reduced resilience. Itraconazole targets ergosterol biosynthesis, and so is expected to affect membrane fluidity. It appears likely that a combination of wall and membrane defects is particularly difficult for a fungal cell to resist. Once UGM inhibitors have been developed, we expect that they will show enhanced efficacy if given in combination with amphotericin B as well as Itraconazole.

Compared to wild type, An*ugmA▵ A. nidulans* walls had higher adhesion to AFM Si_3_N_4_ probe tips [21]. In addition, Af*ugmA▵* strains have been shown to be highly adherent to pulmonary epithelial cells, to glass or plastic surfaces, compared to wild type [15]. Consistent with these previous results, An*umgA▵* and mutant strains have increased hyphal adherence to latex beads compared to wild type strains (Figure 2). Counter-intuitively, higher adhesion [15] has been correlated with both higher and lower pathogenicity [15], [18] but there are few studies of this type and further research is needed. This disparity could be due to lower growth rates in *Af*UgmA mutants, thus potentially also to lower tissue penetration.

Taken together, we have shown that fungal phenotype can provide useful estimates of functional information on the role of some amino acid residues, even though their mutant gene products were poorly expressed *in vitro* (i.e. H63N). Our results in this study suggest that UgmA activity correlated with hyphal phenotype: a<7-fold decrease produced a wild type phenotype (wild type *Af*UgmA, F66A), a ∼20-fold decrease resulted in partial function (H63N and R182K), and a ≥ 70-fold decrease (R182A, R327K, R327A, *An*UgmAΔ) was insufficient to synthesize a functional level of wall Gal*f*.

In conclusion, we have shown that reduced *Af*UgmA activity due to mutation did impair *A. nidulans* growth in a manner substantially similar to gene deletion and gene down-regulation. UgmA distribution was not affected by these manipulations. Loss or absence of Gal*f* increased wall α-glucan but reduced wall β-glucan (which were strongly correlated), and with increased hyphal surface adhesion and Caspofungin sensitivity. Thus, it seems that *A. nidulans* needs a balanced expression of UgmA (not high, not low [13]) to generate a resilient wall for normal growth and wall surface integrity. In the future, we anticipate that our approach will also be useful for assessing the effects of UgmA-targeting drugs (under development by the Sanders group).

## Materials and Methods

### Strains, plasmids, and culture conditions

Strains, primers and plasmids are listed in Table S1 in File S1. The *A. nidulans* strains were maintained on complete medium (CM) supplemented for nutritional markers as described in [27]. Bacterial strains were grown on LB with antibiotics as required (Table S1 in File S1). All water was freshly prepared 18 MegOhm ultrapure (Barnstead NanoDiamond).

### Site-directed mutagenesis and overexpression of F66A-*Af*UGM

Site-directed mutagenesis of the F66A *Af*UGM mutant was performed using the QuikChange^TM^ site-directed mutagenesis kit (Stratagene, Inc.) according to the manufacturer's protocol. Comparable methods were used for creating the other SDM strains [22]. An over-expression vector pET22b harboring the Af*UGM* gene was used as the template DNA. The PCR mixture contained 50 ng of template DNA and 15 pmole of each primer. PCR amplification was carried out in a GeneAmp PCR PTC100 System. The original methylated plasmid was digested with *Dpn*I, then 2 µL of the reaction was used to transform competent *E. coli* Dh5α cells (Novagen). Ampicillin-resistant colonies were selected from the LB plates, and the specific mutation was verified by DNA sequencing. The mutant enzyme was overexpressed and purified as previously described [28].

### Enzyme kinetics

Kinetic constants for F66A *Af*UGM mutant were determined as previously described [22]. A fixed concentration of *Af*UGM mutant protein (500 nM) was chosen so as to have less than 40% conversion to the product UDP-Gal*p*. Reactions were carried out with 0 – 300 µM of UDP-Gal*f* in a final volume of 100 µL 50 mM phosphate buffer pH 7.0 and 20 mM freshly prepared sodium dithionite. The incubations were carried out for 1 min at 37°C then quenched with 100 µL *n*-butanol. The conversion of UDP-Gal*p* to UDP-Gal*f* was monitored at 262 nm using HPLC (Waters). The amount of conversion was determined by integration of the UDP-Gal*p* and UDP-Gal*f* peaks. The initial velocity was calculated from the substrate concentration and percentage UDP-Gal*p* conversion. Kinetic parameters were determined with GraphPad Prism software (GraphPad Software, San Diego, CA) using nonlinear regression analysis.

### Mutagenesis and transformation

Strains used in this study are shown in Table S1 in File S1. The wild type (WT) and wild type complemented (WC) strains were compared with *Af*UgmA mutants R327K, R327A, R182K, R182A, F66A and H63N, and with the An*ugmA*▵ strain.

Af*ugmA* constructs for mutations in the *Af*UgmA active site and in *Af*UgmA loop III that had been used for structural studies [22] were used to generate An*ugmA* replacement constructs using fusion PCR (Figure S3 in File S1) according to [29]. We used an Af*pyrG* selectable marker controlled by the α-tubulin promoter [*tubA*(p)-*pyrG*] to ensure a constitutive level of marker expression, whereas the wild type or mutated version of Af*ugmA* was controlled by their endogenous promoters (Figure S3 in File S1). Constructs were transformed into wild type protoplasts [29], [30]. Confirmation of the correct gene manipulations used genomic DNA (isolated as described in [31]) from putative transformant strains as a template for PCR (Figure S5 and S6 in File S1) with combinations of primers as shown Table S1 in File S1. Prior to phenotype analysis, genomic DNA from each *A. nidulans* strain was extracted. The *ugmA* sequence was amplified from these genomic DNA samples using PCR. Primers that we used to amplify *ugmA* target 100 bp upstream and 100 bp downstream (inside of *tubA* promoter) (Figure S3 in File S1) of *ugmA*. PCR products were sequenced in order to confirm the expected construct generation and replacement. Fusion construction for replacement cassettes used primers that were ∼50 bp inside of upstream and downstream primers from both sides. To confirm the correct insertion site, we used the outside pair of primers.

### Colony growth, sporulation and surface adhesion

Colony characters were examined as in [11]. Strains were streaked on CM and incubated for 3 d at 28 °C to give isolated colonies. The diameter of ten colonies/strain was measured to the nearest millimeter using a dissection microscope. The number of spores produced per colony was counted for four colonies/strain.

The hyphal surface adhesion assay was modified from [15]. Fluorescent (excitation maximum 520 nm; emission maximum 540 nm) 0.5 µm diameter polystyrene beads (Sigma: aqueous suspension, 2.5% solids content) were diluted 1∶10 in sterile phosphate buffered saline (PBS). One hundred microliters of bead solution was added to CM liquid containing 20,000 spores and incubated for 8 h at 37°C with 150 r.p.m. Images of germlings were collected using a Zeiss META510 confocal microscope with a 63x, 1.2 N.A. multi-immersion objective, a 514 nm excitation from Ar ion laser at 20% power, and a 530–600 nm emission filter.

### Cell wall preparation

Cell wall extraction was performed according to [32]. Colonies were grown in shaken liquid at 37°C for 48 h, filtered through Whatman #1 filter paper, washed with ultrapure water, and then with 0.5 M NaCl. Fungal hyphae were broken using 1 mm glass beads in buffer [20 mM Tris, 50 mM EDTA, pH 8.0]. Cell walls were separated from cytoplasmic debris by centrifugation at 3000 x *g* for 10 min. The pellet containing the cell wall fraction was washed with same buffer with stirring for 4 h at 4°C, followed by a wash with ultrapure water. The pellet was frozen at –80°C, then lyophilized overnight.

### ELISA

Our ELISA protocol was adapted from [32]. Isolated *A. nidulans* cell walls [0.5 mg/mL in PBS] were incubated in 96-well Immulon 2HB plates (Sigma) overnight at 4°C. Subsequent steps were performed at room temperature using monoclonal antibodies to Gal*f* (L10; provided Prof. Frank Ebel, Univ Munich), α-1,3-glucan (MOPC-104E; Sigma), and β-glucan [(1-3)-β-glucan directed monoclonal antibody, Cat. No. 400-2, Biosupplies, Australia]. Primary antibodies were diluted 1∶10 (Gal*f*), 1∶30 (α-glucan), or 1∶50 (β-glucan). Secondary antibodies (1∶500) were alkaline phosphatase-conjugated goat anti-mouse IgM (Sigma) (for Gal*f* and α-glucan) and alkaline phosphatase-conjugated goat anti-mouse IgG (for β-glucan). At the final step, wells were incubated with alkaline phosphatase substrate (Sigma) (1 mg/mL) dissolved in substrate buffer (0.5 mM MgCl_2_.6H_2_O, 9.6% diethanolamine, pH 9.6) for 30 min. Absorbance at 405 nm was recorded using an ELISA reader. All ELISA experiments were performed at least twice with three replicates. We used PBS and cell wall extracts of wild type and deleted strains without primary antibody as a control.

### Protein extraction and western blot

To extract total protein, *A. nidulans* conidia were grown in complex media (CM) for 20 h. Mycelia were then collected from liquid cultures by filtration through Miracloth paper, washed with distilled water, dried on paper towels, and ground to a powder with mortar and pestle in liquid nitrogen [33]. The ground mycelium was resuspended and boiled in the urea/SDS buffer [34]: 1% SDS, 9 M urea, 25 mM Tris-HCl (pH 6.8), 1 mM EDTA, and 0.7 M 2 mercaptoethanol). The debris was removed by centrifugation at 13000 x *g*. Total protein concentration was determined by the Bio-Rad protein assay. UgmA-GFP fusions were detected on Western blots using purified mouse anti-GFP polyclonal antibody (eBioscience). IRDye 800-conjugated anti-mouse IgG was used as a secondary antibody. Bands were detected using the Odyssey Infrared Imaging System (LI-COR Biosciences). Images were processed by Odyssey 3.0.16 application software (LI-COR Bioscience).

### Microscopy

Samples were prepared for confocal microscopy as described in [11]. Gal*f*, α-glucan and β-glucan were immunolocalized using the same primary antibodies as described for ELISA. Primary antibodies were used at full strength for L10, at 1∶50 dilutions for α-glucan and β-glucan. GFP was immunolocalized using anti-GFP primary antibody (eBioscience) at a dilution of 1∶100. TRITC-conjugated goat-anti-mouse was used as secondary antibody at 1∶100 dilution. Immunofluorescence procedures followed [13]. Samples were examined using confocal epifluorescence microscopy. The relative immunofluorescence intensity was quantified using Zeiss image browser software at 25 isolated cellular sites at hyphal wall where we had near-median confocal slices of subapical cells.

Scanning electron microscopy was used to examine conidiation following preparation as in [13]. Strains were grown on dialysis tubing laid on CM for 3 d at 28°C. Colonies were fixed by immersion in 1% glutaraldehyde, dehydrated in acetone, critical-point dried (Polaron E3000, Series II), and gold sputter coated (Edwards model S150B). Samples were imaged with a JEOL 840A scanning electron microscope.

### Drug sensitivity testing

Sensitivity to Caspofungin, Itraconazole and Calcofluor White of wild type and engineered UgmA strains was measured using a disc-diffusion assay as described in [12], [13]. Stock solutions: itraconazole (1.6 mg/mL in DMSO), calcofluor white (10 mg/mL in 25 mM KOH), and caspofungin (20 mg/mL in sterile water) were stored at –80°C. For this test, 1×10^7^ spores were mixed into 20 mL of 50°C CM agar and immediately poured in 9 cm Petri plates. Stock solutions were pipetted onto sterile 6 mm paper discs placed on the agar surface: 20 µL of CFW, 10 µL of itraconazole, and 20 µL of caspofungin. Solvent controls, DMSO and 50% ethanol, showed no zone of inhibition. The plates were incubated at 30°C for 48 h, before measuring the zone of inhibition [13].

Drug sensitivity is presented as an index compared to wild type on the same medium, so that values greater than 1.0 indicate hypersensitivity. Statistical analysis of the raw data and comparison to sensitivity indices showed that a difference of >0.2 in index value correlated with statistical significance. Representative plates from the caspofungin study are shown in Figure S7 in File S1. Quantitative results are shown in Table 2.

## Supporting Information

File S1 **Figure S1, Colony morphology and spore colour of** ***Aspergillus nidulans*** **wild type (WT), complemented (WC) strains grown on complete medium at 30°C for 3 d.** Both WT and WC strains were grown on same plate. No difference in colony morphology was observed. Arrow indicates respective strains **Figure S2, Scanning electron micrographs of** ***Aspergillus nidulans*** **showing: colony (uppercase), and conidiophore (lowercase) phenotype for wild type and SDM strains on complex media. Scale bar is 100 µm for colony images and 10 µm for conidiophore images.** WT; wild type (AAE1), WC; complemented with wild type Af*ugmA* (An*ugmA*::Af*ugmA*), F66A; An*ugmA*::Af*ugmA*-F66A, H63N; An*ugmA*::Af*ugmA*-H63N, R182K; An*ugmA*::Af*ugmA*-R182K, R182A; An*ugmA*::Af*ugmA*-R182A, R327K; An*ugmA*::Af*ugmA*-R327K, An*ugmAΔ*. **Figure S3, Strategy for generation of complemented, mutated and GFP-tagged mutated strains in** ***A. nidulans*** **.** A. Replacement of *A. nidulans ugmA* with wild type and mutated *A. fumigatus ugmA*. B. GFP-tagging of *A. nidulans* UgmA with wild type and mutated *A. fumigatus* UgmA. **Figure S4, GFP immunolocalization in** ***Aspergillus nidulans*** **using anti-GFP antibody.** Wild type complemented (WC) and single residue mutants (H63N, R182A and R327A) have GFP localization comparable to *Af*UgmA-GFP distribution. Green channel: GFP distribution; Red channel: anti-GFP straining. Bar  =  20 µm for all images. **Figure S5, Confirmatory PCR for SDM replacement strain.** This figure is for replacement of An*ugmA* by Af*ugmA*-R327A in *A. nidulans*. In addition to confirmatory PCR, confirmation of all SDM strains was also done by DNA sequencing. **Figure S6, Confirmatory PCR for SDM-GFP tagging strains.** This figure is for tagging Af*ugmA*-R327A with GFP and expressing in *A. nidulans*. In addition to Confirmatory PCR, confirmation of all GFP tagged strains was also done by DNA sequencing. **Figure S7, Response of wild type, complemented, mutated and deleted strains to Caspofungin.** Sensitivity is measured at the innermost clear zone (arrows). WT; wild type, An*ugmA*::Af*ugmA*, An*ugmA*::Af*ugmA*-F66A, An*ugmA*::Af*ugmA*-H63N, An*ugmA*::Af*ugmA*-R182K, An*ugmA*::Af*ugmA*-R182A, An*ugmA*::Af*ugmA*-R327K, An*ugmA*::Af*ugmA*-R327A, An*ugmAΔ*. **Table S1, Strains and primers used in this study.**
(DOC)
