# Supplementary material for: Aspergillus nidulans Cell Wall Composition and Function Change in Response to Hosting Several Aspergillus fumigatus UDP-Galactopyranose Mutase Activity Mutants
Source: PLoS One. 2014 Jan 15;9(1):e85735. doi: 10.1371/journal.pone.0085735 (PMC3893270; doi:10.1371/journal.pone.0085735)
Supplement: File S1 — Figure S1, Colony morphology and spore colour of Aspergillus nidulans wild type (WT), complemented (WC) strains grown on complete medium at 30°C for 3 d. Both WT and WC strains were grown on same plate. No difference in colony morphology was observed. Arrow indicates respective strains Figure S2, Scanning electron micrographs of Aspergillus nidulans showing: colony (uppercase), and conidiophore (lowercase) phenotype for wild type and SDM strains on complex media. Scale bar is 100 µm for colony images and 10 µm for conidiophore images. WT; wild type (AAE1), WC; complemented with wild type AfugmA (AnugmA::AfugmA), F66A; AnugmA::AfugmA-F66A, H63N; AnugmA::AfugmA-H63N, R182K; AnugmA::AfugmA-R182K, R182A; AnugmA::AfugmA-R182A, R327K; AnugmA::AfugmA-R327K, AnugmAΔ. Figure S3, Strategy for generation of complemented, mutated and GFP-tagged mutated strains in A. nidulans . A. Replacement of A. nidulans ugmA with wild type and mutated A. fumigatus ugmA. B. GFP-tagging of A. nidulans UgmA with wild type and mutated A. fumigatus UgmA. Figure S4, GFP immunolocalization in Aspergillus nidulans using anti-GFP antibody. Wild type complemented (WC) and single residue mutants (H63N, R182A and R327A) have GFP localization comparable to AfUgmA-GFP distribution. Green channel: GFP distribution; Red channel: anti-GFP straining. Bar = 20 µm for all images. Figure S5, Confirmatory PCR for SDM replacement strain. This figure is for replacement of AnugmA by AfugmA-R327A in A. nidulans. In addition to confirmatory PCR, confirmation of all SDM strains was also done by DNA sequencing. Figure S6, Confirmatory PCR for SDM-GFP tagging strains. This figure is for tagging AfugmA-R327A with GFP and expressing in A. nidulans. In addition to Confirmatory PCR, confirmation of all GFP tagged strains was also done by DNA sequencing. Figure S7, Response of wild type, complemented, mutated and deleted strains to Caspofungin. Sensitivity is measured at the innermost clear zone (arrows). WT; wild type, Anugm [file pone.0085735.s001.doc]

Table S1: Strains and primers used in this study

***Aspergillus***

A1100 ^a^ *Aspergillus fumigatus* wild type, AF293

A1149 ^a^  *Aspergillus nidulans* *pyrG89*; *pyroA4*; *nkuA*::*argB*

AAE1 *Aspergillus nidulans* *pyrG89*:: Nc*pyr4+;* *pyroA4*; *nkuA*::*argB*

*A. nidulans ugmA*Δ ^b^ AN3112::Af*pyrG*; *pyrG89*; *pyroA4*; *nkuA*::*argB*

An*ugmA*::Af*ugmA*^c^ AN3112::AfugmA:*tubA*p:Af*pyrG*; *pyrG89; pyroA4; nkuA::argB*

F66A^c^ AN3112::AfugmA-F66A:*tubA*p:Af*pyrG*;*pyrG89;pyroA4;nkuA::argB*

H63N^c^ AN3112::AfugmA-H63N:*tubA*p:Af*pyrG*;*pyrG89;pyroA4;nkuA::argB*

R182K^c^ AN3112::AfugmA-R182K:*tubA*p:Af*pyrG*;*pyrG89;pyroA4;nkuA::argB*

R182A^c^ AN3112::AfugmA-R182A:tubAp:Af*pyrG*;*pyrG89;pyroA4;nkuA::argB*

R327K^c^ AN3112::AfugmA-R327K:*tubA*p:Af*pyrG*;*pyrG89;pyroA4;nkuA::argB*

R327A^c^ AN3112::AfugmA-R327A:*tubA*p:Af*pyrG*;*pyrG89;pyroA4;nkuA::argB*

Af*ugmA*-GFP^c^ AN3112::AfugmA-GA5-GFP-*tubA*p-Af*pyrG*;*pyrG89;pyroA4; nkuA::argB*

H63N-GFP^c^ AN3112::Af*ugmA*-H63N-GA_5_-GFP-*tubA*p-Af*pyrG*;*pyrG89*;*pyroA4*;

*nkuA*::*argB*

R182A-GFP^c^ AN3112::Af*ugmA*-R182A-GA5-GFP-*tubA*p-Af*pyrG*;*pyrG89*;*pyroA4*;

*nkuA*::*argB*

R327A-GFP^c^ AN3112::Af*ugmA*-R327A-GA5-GFP-*tubA*p-Af*pyrG*;*pyrG89*;*pyroA4*;

*nkuA*::*argB*

***Escherichia coli***

BL21-gold (DE3) ^d^

**Plasmids**

pAf*ugmA* ^e^ pET22b, Af*ugmA*, amp^R^

pAf*ugmA*-F66A ^e^ pET22b, Af*ugmA*-F66A, amp^R^

pAf*ugmA*-H63N ^e^ pET22b, Af*ugmA*-H63N, amp^R^

pAf*ugmA*-R182K ^e^ pET22b, Af*ugmA*-R182K, amp^R^

pAf*ugmA*-R182A ^e^ pET22b, Af*ugmA*-R182A, amp^R^

pAf*ugmA*-R327K ^e^ pET22b, Af*ugmA*-R327K, amp^R^

pAf*ugmA*-R327A ^e^ pET22b, Af*ugmA*-R327A, amp^R^

pFNO3 ^a^ GA5-GFP, Af*pyrG*, Kan^R^

**Primers**  Sequence 5’ 🡪 3’

Replacement of An*ugmA*

P1, Up An*ugmA* F^c^ GACTCTTGAGATTTGCTTGGGT

P2, Up AnugmA R^c^ ACATATCGGGGTGGGTCATGAAGAGAGCGAAGCTGCAG

P3, Af*ugmA* F ^c^ ATGACCCACCCCGATATGT

P4, Af*ugmA*(*tupA*p-tail) R^c^ AGTCACGTGCTGCATTTACTGGGCCTTGCTCTTG

P5, *tupA*p F ^c^ ATGCAGCACGTGACTATT

P6, *tupA*p (*pyrG*-tail) R ^c^ AATTGCGACTTGGACGACATCTTGTCTAGGTGGGTGGT

P7, Af*pyrG* F^c^ ATGTCGTCCAAGTCGCAATT

P8, Af*pyrG* R^c^ TCATGACTTGCCGCATACTC

P9, dn An*ugmA*(*pyrG*-T) F^c^ GAGTATGCGGCAAGTCATGAAACTCTTCTGCGTGGATGG

P10, dn An*ugmA* R^c^ GGACTGCAGGTTGAAGCAG

Fusion construct generation for replacement

P11, Fusion Up An*ugmA* F^c^ CCGTCCTTCGTAGAGTACTTGAG

P12, Fusion dnAn*ugmA* R^c^ GGTTGAACGATGTCAGCGTA

Replacement confirmation of An*ugmA*

P1, Up An*ugmA* F^c^ GACTCTTGAGATTTGCTTGGGT

P10, dn An*ugmA* R^c^ GGACTGCAGGTTGAAGCAG

Ame8, mid*PyrG* R^b^ CACATCCGACTGCACTTCC

Af*ugmA*-*gfp* tagging

P13, Af*ugmA*(GFP-tail) R ^c^ CTCCAGCGCCTGCACCAGCTCCCTGGGCCTTGCTCTTGG

P14, Dn An*ugmA* R ^c^ ATCAGTGCCTCCTCTCAGACAGAACTCTTCTGCGTGGATGG

Ame 27 5GA-GFPF^b^ GGAGCTGGTGCAGGC

Ame 28 5GA-GFPR^b^ TCATGACTTGCCGCATACT

Af*ugmA*-*gfp* tagging confirmation

P1, Up An*ugmA* F^c^ GACTCTTGAGATTTGCTTGGGT

P10, dn An*ugmA* R^c^ GGACTGCAGGTTGAAGCAG

Ame8, mid*PyrG* R^b^ CACATCCGACTGCACTTCC

Fusion constructs generation using fusion PCR for GFP tagging strains (same as replacement)

P11, Fusion Up An*ugmA* F^c^ CCGTCCTTCGTAGAGTACTTGAG

P12, Fusion dnAn*ugmA* R^c^ GGTTGAACGATGTCAGCGTA

H63N SDM

H63N forward GTCGGTGGTCACGTCATCGCCTCCCACTACAAGTATTTC

H63N reverse GAAATACTTGTAGTGGGAGGCGATGACGTGACCACCGAC

a Fungal Genetics Stock Center, www.fgsc.net

b El-Ganiny et al. (2008)

c This study

d Novagen (www.emdchemicals.com)

e van Straaten et al. (2012)


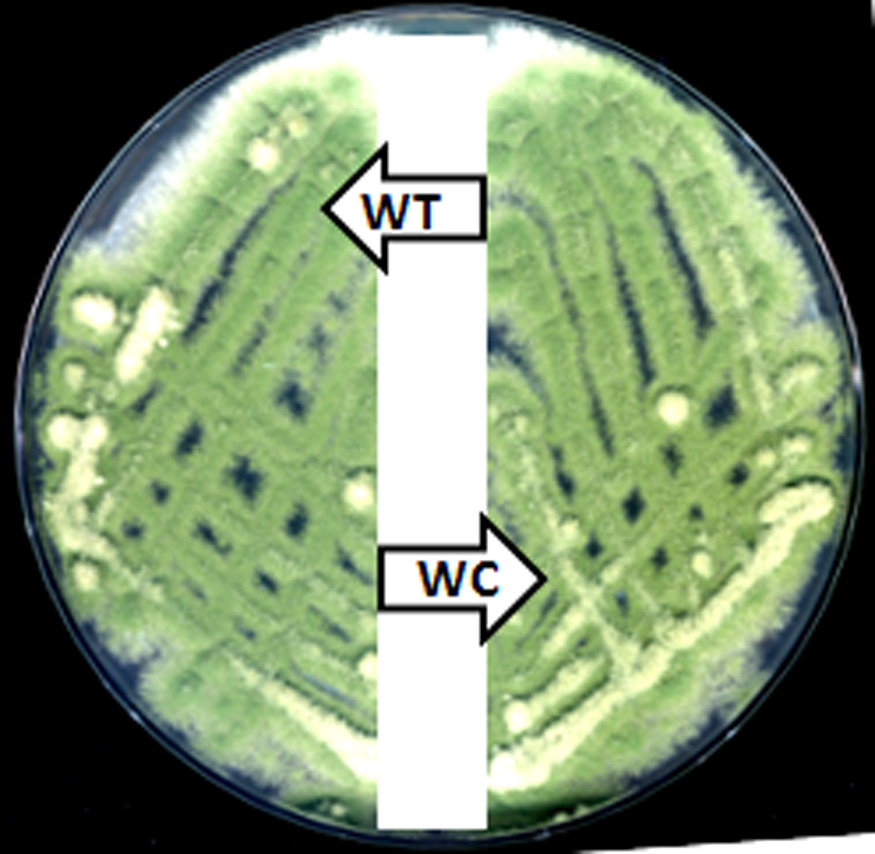


Figure S1: Colony morphology and spore colour of *Aspergillus nidulans* wild type (WT), complemented (WC) strains grown on complete medium at 30 °C for 3 d. Both WT and WC strains were grown on same plate. No difference in colony morphology was observed. Arrow indicates respective strains.

***
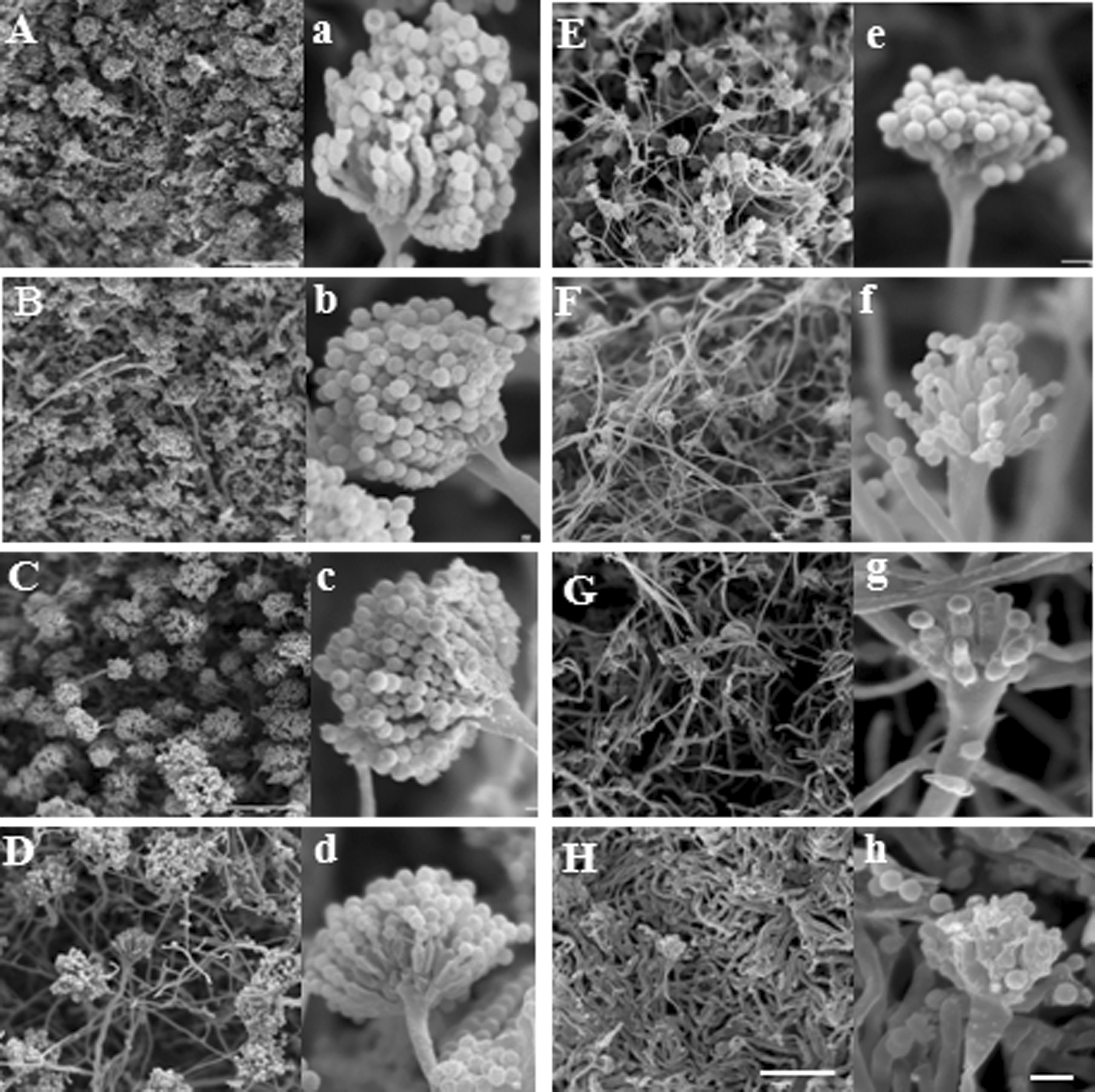
***

**Figure S2.** Scanning electron micrographs of *Aspergillus nidulans* showing: colony (A-H), and conidiophore (a-h) phenotype for wild type and mutated strains on complex media. Scale bar is 100 μm for A -H and 10 μm for a-h. A) wild type (AAE1), B) An*ugmA*::Af*ugmA*, C) An*ugmA*::Af*ugmA-*F66A, D) An*ugmA*::Af*ugmA*-H63N, E) An*ugmA*::Af*ugmA*-R182K, F) An*ugmA*::Af*ugmA*-R182A, G) An*ugmA*::Af*ugmA*-R327K, H) An*ugmA*::Af*ugmA*-R327A, I) An*ugmA*Δ.


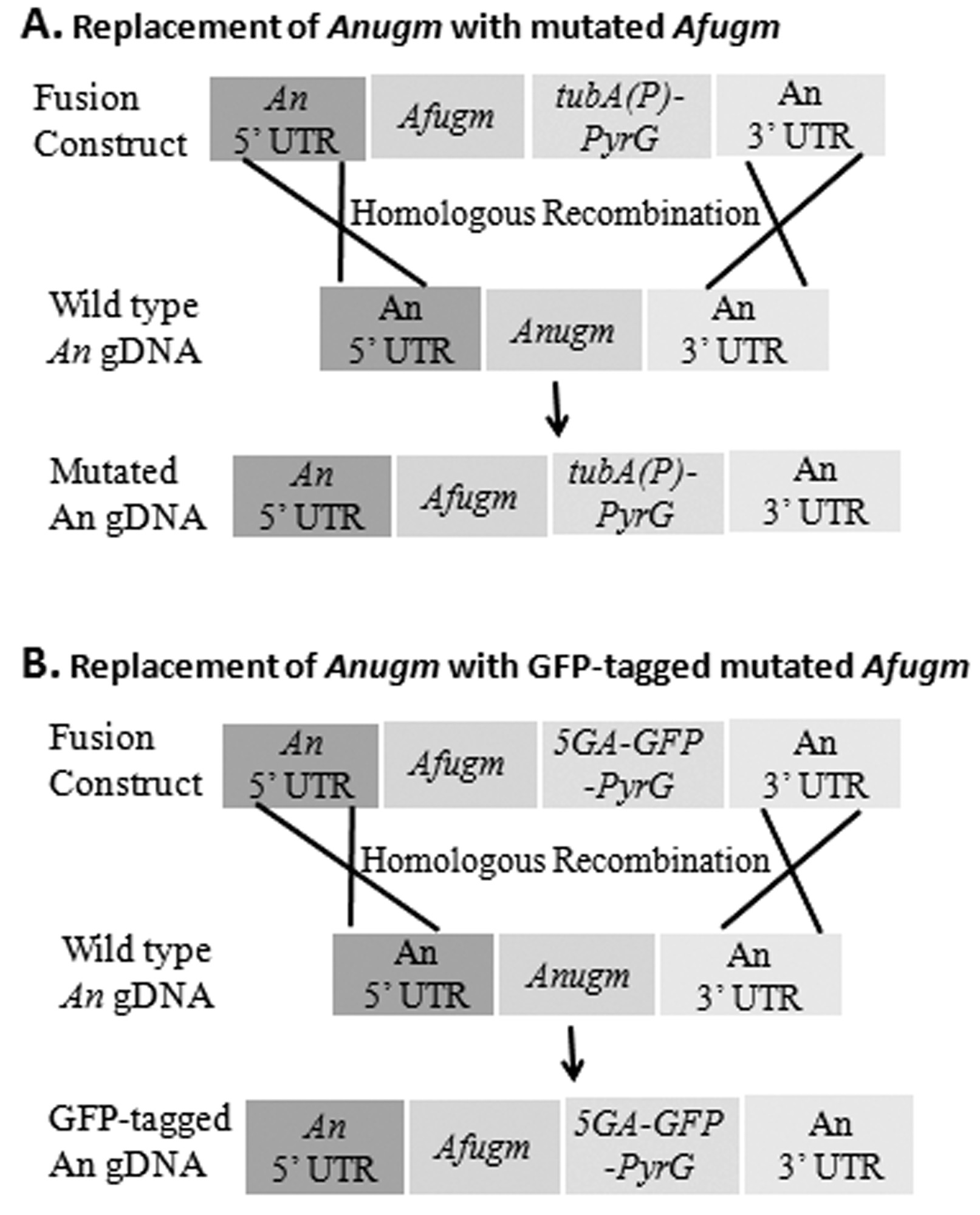


**Figure S3**. Strategy for generation of complemented, mutated and GFP tagged mutated strains in *A. nidulans.* A. Replacement of *A. nidulans* *ugmA* with wild type and mutated *A. fumigatus* *ugmA*. B. GFP-Tagging of *A. nidulans* UgmA with wild type and mutated *A. fumigatus* UgmA*.*


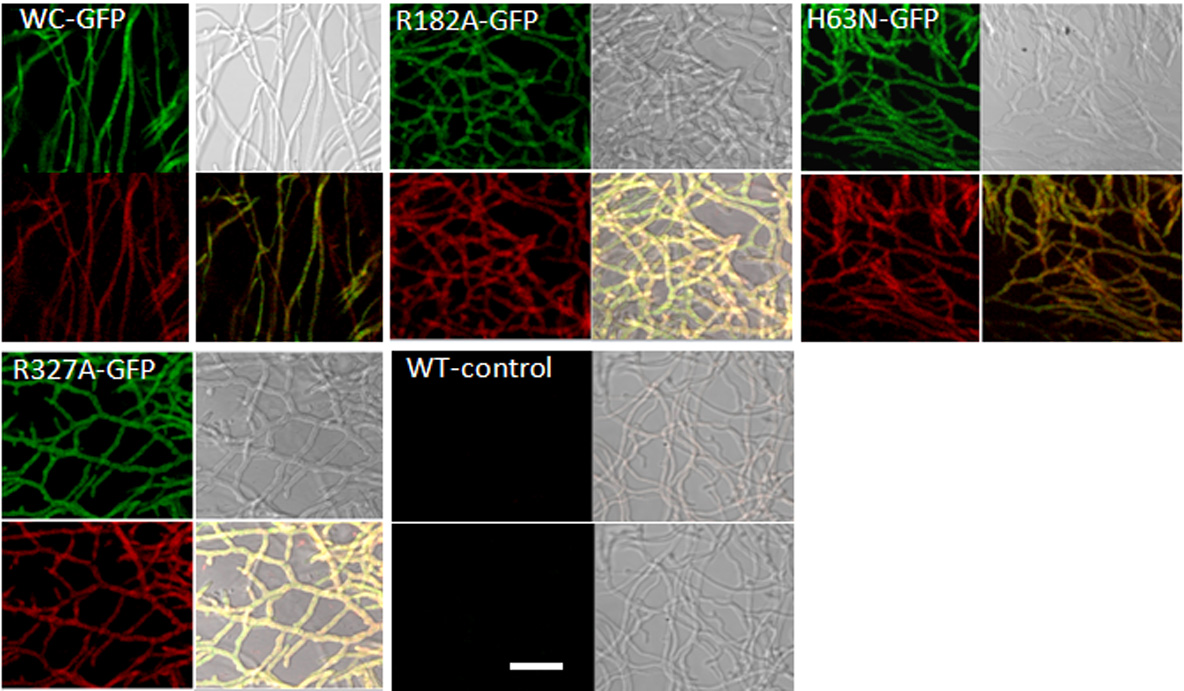


**Figure S4.** GFP immunolocalization in *Aspergillus nidulans* using anti-GFP antibody. Wild type complemented (WC) and single residue mutants (H63N, R182A and R327A) have GFP localization comparable to *Af*UgmA-GFP distribution. Green channel: GFP distribution; Red channel: anti-GFP straining. Bar = 20 µm for all images.


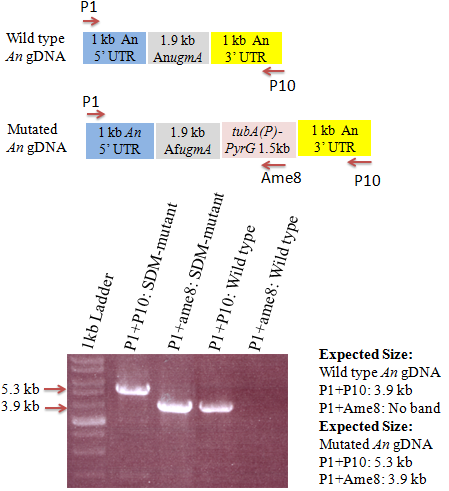


**Figure S5.** Confirmatory PCR for site directed mutated (SDM) replacement strain. This figure is for replacement of An*ugmA* by Af*ugmA*-R327A in *A. nidulans*. In addition to Confirmatory PCR, confirmation of all SDM strains was also done by DNA sequencing.


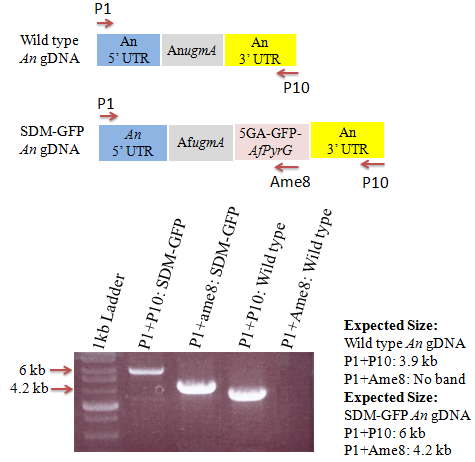


**Figure S6.** Confirmatory PCR for SDM-GFP tagging strains. This figure is for tagging Af*ugmA*-R327A with GFP and expressing in *A. nidulans***.** In addition to Confirmatory PCR, confirmation of all GFP tagged strains was also done by DNA sequencing.


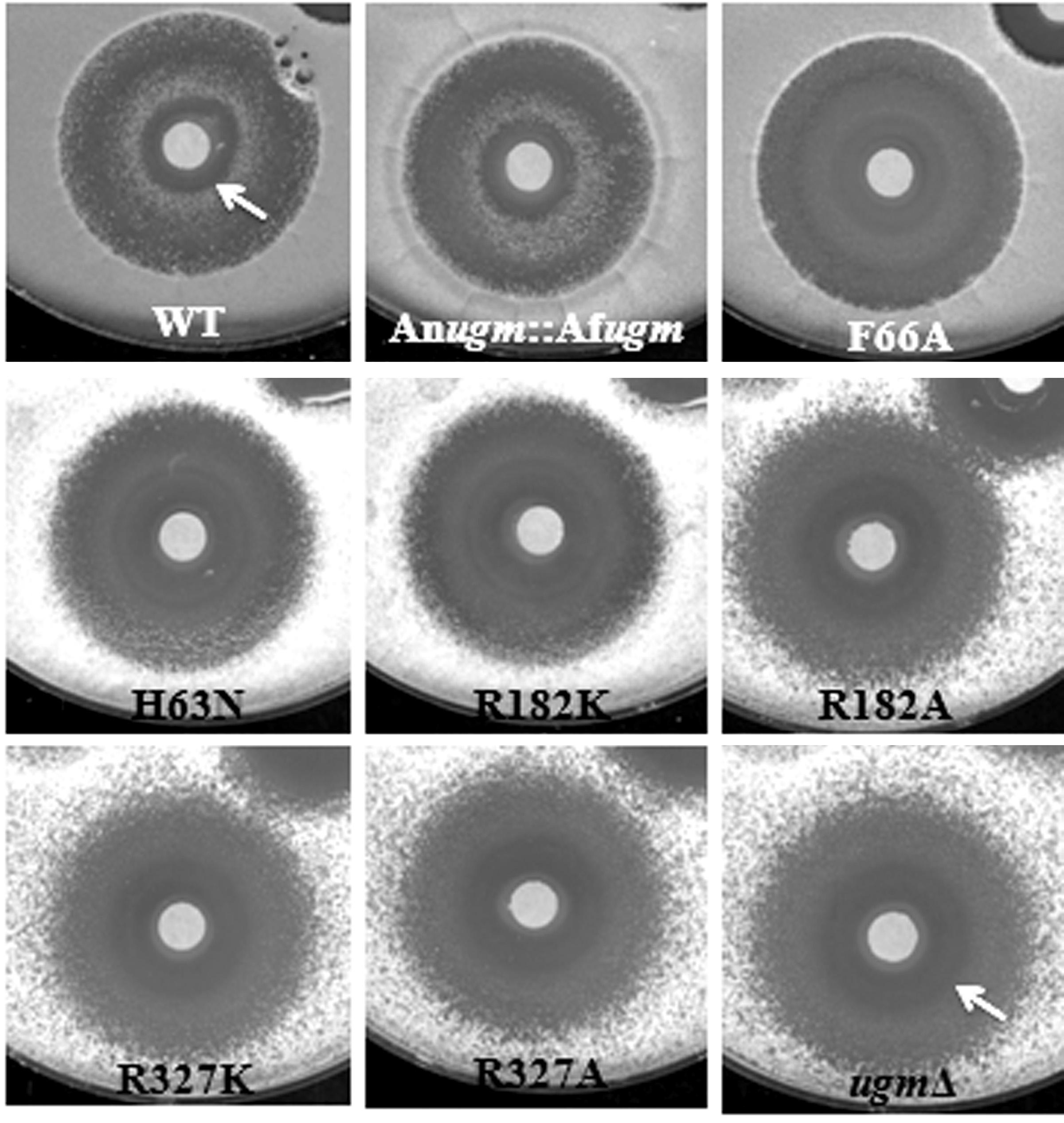


**Figure S7:** Response of wild type (WT), complemented, mutated and deleted strains to Caspofungin. Sensitivity is measured at the innermost clear zone (arrows). WT; wild type, An*ugmA*::Af*ugmA*, An*ugmA*::Af*ugmA-*F66A, An*ugmA*::Af*ugmA*-H63N, An*ugmA*::Af*ugmA*-R182K, An*ugmA*::Af*ugmA*-R182A, An*ugmA*::Af*ugmA*-R327K, An*ugmA*::Af*ugmA*-R327A, An*ugmA*Δ.
